# Biomechanical evaluation of narrow-diameter Ti–Zr implant systems at different lingual inclination angles in the molar region

**DOI:** 10.3389/froh.2026.1878801

**Published:** 2026-07-06

**Authors:** Lishan Li, Zhe Chen, Jinchuan Zheng, Hang Lin, Wenbeng Xie

**Affiliations:** 1 People’s Hospital Affiliated of Quanzhou Medical College, Fujian, China; 2 Department of Oromaxillofacial Head and Neck Surgery, Center for Oromaxillofacial Head and Neck Disorders, Huashan Hospital, Fudan University, Shanghai, China; 3 Department of Oral Maxillo-Facial Surgery, the First Affiliated Hospital, Fujian Medical University, Fuzhou, China

**Keywords:** inclined implantation, *in-vitro* experiment, molar region, narrow-diameter implant, oblique loading, three-dimensional finite element analysis, titanium–zirconium alloy

## Abstract

**Background:**

Narrow-diameter implants may be considered in posterior sites with limited bone volume, while lingual inclination may help avoid anatomical constraints. However, their biomechanical behavior under different loading directions remains unclear.

**Objective:**

To compare the system-level biomechanical responses of a 3.3 × 12 mm titanium–zirconium implant configuration and a 4.1 × 10 mm commercially pure titanium implant configuration at different lingual inclination angles.

**Methods:**

Three-dimensional finite element models were constructed for both implant systems at 0°, 5°, 10°, 15°, and 20°. A 150 N vertical load and a 150 N load applied at 30° to the prosthetic crown axis were simulated. Implant von Mises stress and cortical and cancellous bone maximum principal stress were evaluated. Formal mesh convergence analysis was performed using three mesh densities. Exploratory fatigue analysis and *in vitro* displacement testing were conducted under vertical loading.

**Results:**

Mesh convergence showed less than 5% change between the medium and fine meshes for all primary stress outcomes. Under vertical loading, implant stress decreased from 0° to 10° and then showed modest fluctuations. Oblique loading increased implant and peri-implant bone stresses in all configurations, with generally greater amplification at larger inclination angles. Implant stress remained lower at 10° than at 0° under both loading conditions, whereas cortical bone stress increased across the same range. *In vitro* displacement remained relatively stable from 0° to 10° and increased at 15° and 20°.

**Conclusions:**

Inclination-related responses depended on loading direction and the evaluated biomechanical outcome. The 0°–10° range should not be interpreted as uniformly favorable or clinically recommended. Because the systems differed in material, diameter, length, and geometry, the findings represent system-level comparisons.

## 1 Introduction

Dental implant therapy has become a widely used treatment option for patients with partial or complete edentulism (1). A key requirement for predictable implant treatment is stable osseointegration, which depends on sufficient contact between the implant surface and the surrounding alveolar bone. However, tooth loss is frequently followed by alveolar bone resorption, leading to reduced ridge width, altered ridge morphology, and unfavorable relationships with anatomical structures (2). In posterior regions, insufficient bone volume may complicate implant placement and increase the need for additional surgical procedures (3).

Horizontal or vertical bone augmentation can improve implant placement conditions, but these procedures may increase surgical trauma, treatment complexity, patient discomfort, and complication risk (3). Inclined implant placement has therefore been proposed as an alternative strategy in selected cases. By tilting the implant mesiodistally or buccolingually, clinicians may avoid vital anatomical structures and increase the available implant length and bone contact area (4). Nevertheless, implant inclination also changes the direction of load transfer at the implant–bone interface and may increase stress concentration in the crestal cortical bone, particularly when the available buccolingual bone width is limited (5–7).

Peri-implant bone thickness, especially preservation of the buccal and lingual cortical plates, is important for primary stability and favorable peri-implant tissue conditions. Insufficient cortical bone thickness may compromise load transfer and increase the risk of marginal bone remodeling. These biomechanical concerns are particularly relevant for narrow-diameter implants, which have a reduced cross-sectional area and may be more susceptible to stress concentration at the implant neck and surrounding cortical bone under posterior loading conditions (8, 9). Finite element analysis (FEA) is commonly used to investigate these biomechanical interactions because it allows controlled evaluation of implant geometry, material properties, bone conditions, prosthetic design, connection type, and loading direction. A recent systematic review by Azhdari et al. highlighted that peri-implant stress distribution should be interpreted as the result of interactions among implant design, bone quality, and loading configuration rather than as the effect of a single isolated factor (10, 11).

Reducing implant diameter may help preserve peri-implant bone thickness in anatomically constrained sites; however, smaller diameter may also increase mechanical demand on the implant structure. Titanium–zirconium (Ti–Zr) alloys have been introduced to address some of these concerns because they provide higher mechanical strength and a relatively lower elastic modulus than commercially pure titanium. In addition to their mechanical properties, Zr incorporation may improve electrochemical stability and corrosion resistance through the formation of a stable passive oxide layer, which may help maintain implant surface integrity under long-term functional loading (12, 13). Previous studies have suggested that Ti–Zr alloys containing approximately 13%–15% zirconium have favorable properties for dental implant applications (14), and clinical studies have reported comparable short-term performance between narrow-diameter Ti–Zr implants and standard-diameter titanium implants in selected indications (15, 16).

Despite these potential advantages, the biomechanical behavior of narrow-diameter Ti–Zr implant systems placed at different lingual inclination angles in the molar region remains insufficiently understood. Existing FEA studies have provided valuable information regarding implant diameter, length, macrogeometry, connection design, material properties, bone quality, and loading direction; however, relatively few studies have focused on the combined biomechanical effects of narrow-diameter Ti–Zr implant configurations and lingual inclination in posterior high-load regions (17). Because implant inclination, implant dimensions, material properties, and prosthetic alignment may interact with each other, the mechanical response should be evaluated at the implant-system level.

Therefore, the present study used three-dimensional finite element analysis, *in vitro* displacement testing, and exploratory fatigue estimation to compare the system-level biomechanical responses of a narrow-diameter 3.3 × 12 mm Ti–Zr implant configuration and a 4.1 × 10 mm commercially pure titanium implant configuration at different lingual inclination angles in the molar region. Because the two implant systems differed in diameter, length, material, and geometry, the study was not designed to isolate the independent effects of Ti–Zr alloy, implant diameter, implant length, or macrogeometry. Rather, it was designed as a simplified and exploratory comparison of complete implant-system configurations under standardized loading and inclination conditions. The findings were not intended to determine clinical feasibility, define a clinically acceptable inclination threshold, or support surgical recommendations.

## 2 Materials and methods

### 2.1 Establishment of the three-dimensional finite element models

A simplified right mandibular bone block measuring 15 mm (mesiodistal) × 15 mm (buccolingual) × 25 mm (occlusoapical) was constructed using SolidWorks (Dassault Systèmes, France). Cortical bone with a thickness of 2 mm was generated using the shell command, and the internal region was defined as cancellous bone. This simplified segmental mandibular model was used to enable standardized comparisons among implant inclination conditions.

Two clinically relevant implant-system configurations based on the Straumann bone-level implant system were evaluated: a 3.3 × 12 mm titanium–zirconium (Ti–Zr) implant and a 4.1 × 10 mm commercially pure titanium implant (Straumann, Switzerland). These implant dimensions were selected because their nominal lateral surface areas were comparable when estimated using a cylindrical approximation. The estimated lateral surface areas were approximately 124.4 mm² for the 3.3 × 12 mm implant and 128.8 mm² for the 4.1 × 10 mm implant, corresponding to a difference of approximately 3.4%. These values represent nominal cylindrical estimates rather than the actual surface areas of threaded implants, which may be influenced by thread design, taper, connection geometry, and surface roughness. The implant geometries were reconstructed using ANSYS SpaceClaim (ANSYS Inc., USA) and Creo Parametric (PTC Inc., USA). The two configurations were evaluated as complete implant systems rather than as a factorial design intended to isolate the independent effects of material, diameter, length, or macrogeometry.

**Figure 1:**
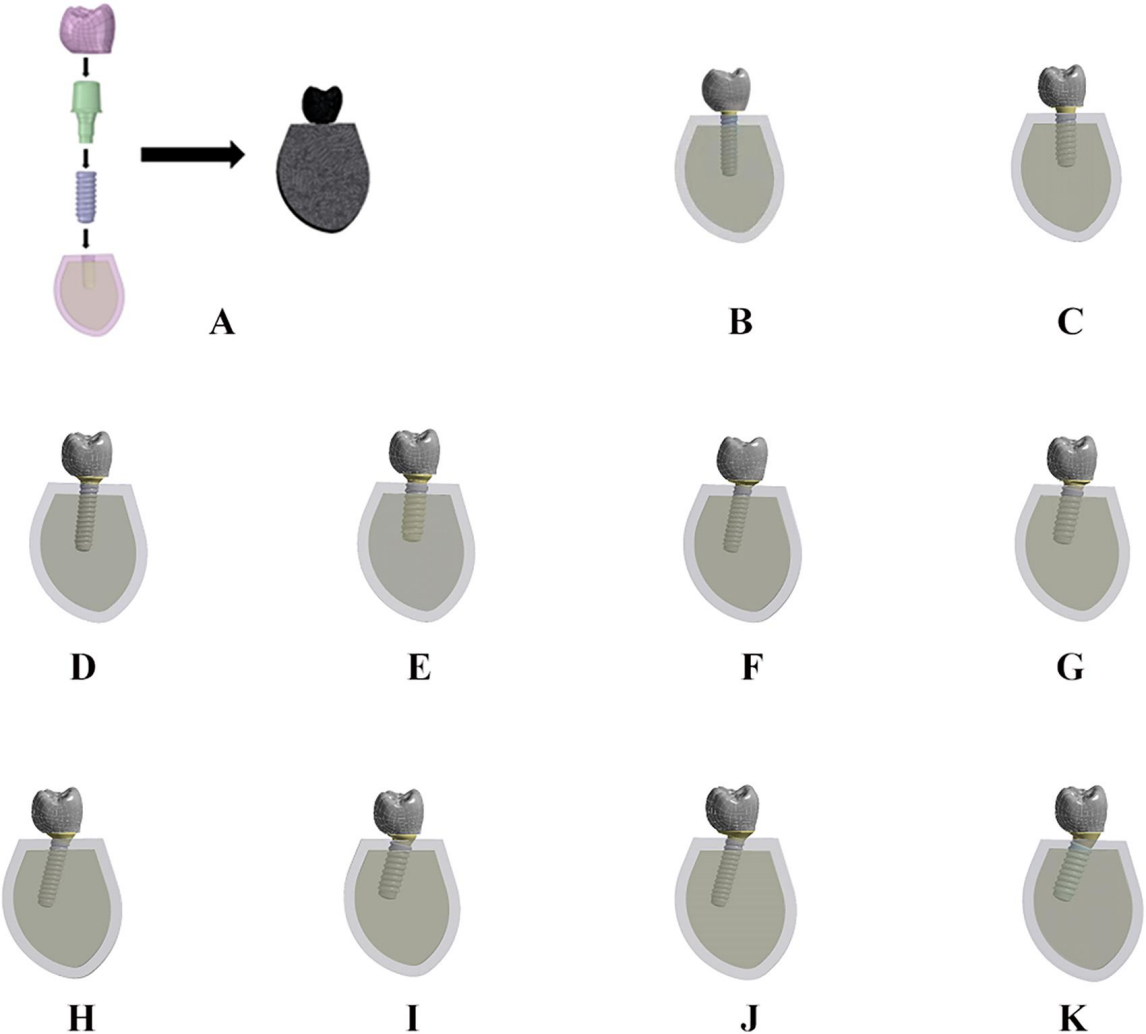
**(A)** Schematic illustration of the model assembly. **(B)** and **(C)** Model with the implant positioned at 0° lingual inclination. **(D)** and **(E)** Model with the implant positioned at 5° lingual inclinations. **(F)** and **(G)** Model with the implant positioned at 10° lingual inclinations. **(H)** and **(I)** Model with the implant positioned at 15° lingual inclinations. **(J)** and **(K)** Model with the implant positioned at 20° lingual inclinations.

Angled abutments corresponding to lingual inclination angles of 0°, 5°, 10°, 15°, and 20° were designed using Exocad (Exocad GmbH, Germany). Crown geometries generated in Exocad were processed in Geomagic Wrap (Geomagic Inc., USA) by smoothing, noise reduction, and remeshing. The implant, abutment, and crown components were assembled in ANSYS SpaceClaim. Implant osteotomies were created by Boolean subtraction, and each implant was positioned such that the most coronal point of the implant neck was level with the bone crest. The abutments were adjusted so that the long axis of the prosthetic crown remained parallel to the long axis of the mandibular block at all inclination angles. In total, ten finite element models were generated, comprising five inclination angles for each implant system (Figure 1).

### 2.2 Material properties and contact definitions

The assembled models were imported into ANSYS 2020R2 for finite element analysis. The mandibular bone block was defined as type II bone according to the Lekholm and Zarb classification (18). The 3.3 × 12 mm implant was assigned Ti–Zr alloy properties corresponding to a commercially available Roxolid-type alloy composed of approximately 85% titanium and 15% zirconium, whereas the 4.1 × 10 mm implant was assigned commercially pure titanium properties. The abutments were defined as titanium, and the crowns were defined as zirconia. The elastic modulus and Poisson's ratio values of all components were obtained from the literature and are listed in Table 1 (19–22).

**Table 1:** Material properties of each component.

Materials	Elasticity modulus(GPa)	Poisson's ratio
Ti–Zr	100	0.30
Ti	110	0.35
Zirconia	200	0.31
Cortical bone	13.70	0.30
Cancellous bone	1.37	0.30

All materials were modeled as homogeneous, isotropic, and linearly elastic to improve numerical stability and permit standardized comparisons among models. The implant–bone interface was defined as fully bonded to represent ideal osseointegration. The implant–abutment and abutment–crown interfaces were also defined as bonded contacts to reduce contact-related nonlinearities and focus on the comparative biomechanical response of the implant–bone system. These assumptions represent simplified model conditions and were considered when interpreting the results.

### 2.3 Mesh generation and convergence analysis

Automatic meshing was performed in ANSYS 2020R2 using three-dimensional tetrahedral solid elements for the crown, abutment, implant, cortical bone, and cancellous bone components. Automatic tetrahedral meshing was selected because the implant–abutment–crown assemblies contained complex curved surfaces, thread-related geometric features, and multiple material interfaces. This approach enabled consistent mesh generation across models while permitting local refinement in regions with expected high stress gradients.

Local mesh refinement was applied at the implant neck, implant–abutment junction, implant–bone interface, cervical cortical bone, and buccal coronal one-third of the implant. Regions distant from the implant were assigned a relatively coarser mesh because lower stress gradients were expected. Mesh quality was evaluated before solving using the element-quality indicators available in ANSYS, including skewness, aspect ratio, and Jacobian-related measures. The same meshing algorithm, model geometry, material properties, contact definitions, boundary conditions, loading conditions, and stress-extraction regions were maintained across mesh densities.

A formal mesh convergence analysis was performed using representative 0° and 20° configurations from both implant systems. These configurations were selected to represent the lower and upper limits of the investigated inclination range. For each representative model, coarse, medium, and fine meshes were generated by progressively reducing the global and local element sizes while retaining the same automatic tetrahedral meshing strategy and local refinement regions.

The primary outcomes used for convergence assessment were implant peak von Mises stress, cortical bone maximum principal stress, and cancellous bone maximum principal stress. The percentage change between successive mesh densities was calculated as follows:

Percentagechange=|Result_finer−Result_coarser|/Result_finer×100%.

Mesh convergence was considered acceptable when the percentage change between the medium and fine meshes was less than 5% for the primary stress outcomes. The final fine meshes contained approximately 1.46–1.51 million nodes and 1.03–1.06 million elements across the ten models. Detailed convergence results are provided in Supplementary Table S1.

### 2.4 Boundary and loading conditions

The inferior surface of the mandibular bone block was fixed to provide a stable reference boundary in the simplified segmental model. This boundary condition was applied consistently across all models to permit direct comparison among inclination angles and implant systems.

For the reference loading condition, a static vertical load of 150 N was applied to the central fossa of the crown parallel to the prosthetic crown axis rather than to the inclined implant axis. This load magnitude was selected to represent a simplified functional occlusal loading condition for implant-supported restorations and to permit standardized comparison among models (23–25).

To evaluate whether the inclination-related biomechanical response remained consistent under a representative non-axial loading condition, an additional static load of 150 N was applied at 30° to the prosthetic crown axis in the buccolingual plane at the same crown central-fossa loading point. The oblique load was directed from the lingual toward the buccal aspect and consisted of an axial component of approximately 129.9 N and a horizontal component of 75.0 N. All model geometry, material properties, contact definitions, mesh settings, boundary conditions, and outcome-extraction regions were kept unchanged between the vertical and oblique loading conditions. The additional oblique loading condition was intended as a representative static sensitivity analysis rather than as a complete reproduction of posterior mastication. It did not incorporate changing occlusal contact locations, mesiodistal loading components, lateral excursions, parafunctional loading, impact, or time-dependent variations during the masticatory cycle.

### 2.5 Stress calculation and outcome measures

The models were solved under both the vertical and oblique loading conditions. Von Mises stress was calculated for the implant components to evaluate equivalent stress distribution in the metallic implant structures. For peri-implant bone, maximum principal stress was used as the primary stress outcome. In this study, maximum principal stress refers to the largest positive principal stress value, representing tensile stress within the cortical and cancellous bone regions. This outcome was selected because tensile stress concentration in peri-implant cortical bone is considered relevant to overload-related bone remodeling and potential marginal bone loss.

Cortical and cancellous bone stresses were extracted separately from predefined peri-implant regions. Cortical bone stress was extracted from the 2 mm cortical shell surrounding the implant, particularly the coronal peri-implant cortical region adjacent to the implant neck. Cancellous bone stress was extracted from the internal cancellous bone region in contact with the implant surface beneath the cortical shell. The same extraction regions were used consistently for all inclination angles, both implant systems, and both loading conditions.

The primary finite element outcomes were implant peak von Mises stress, cortical bone maximum principal stress, and cancellous bone maximum principal stress. These outcomes were compared across inclination angles and between the vertical and oblique loading conditions.

### 2.6 Fatigue analysis

Fatigue behavior was estimated from the finite element stress results obtained under the simplified vertical cyclic loading protocol using ANSYS nCode DesignLife. The implant body was defined as the fatigue-critical component, and fatigue damage was evaluated in regions with high stress concentration, particularly the implant neck, implant–abutment junction, and buccal coronal one-third region. A stress–life approach based on S–N data was used to estimate the number of cycles to failure for each implant system and inclination angle.

A simplified cyclic loading protocol consisted of a loaded phase under 150 N followed by an unloaded phase. Because implant-specific S–N data for Ti–Zr alloy are limited, the fatigue parameters were estimated using available published mechanical-property data and the material-estimation approach implemented in ANSYS nCode DesignLife (19, 26, 27). Accordingly, the fatigue analysis was considered exploratory and hypothesis-generating. The fatigue outputs were used only to describe relative fatigue tendencies among models under identical simplified numerical assumptions and were not interpreted as quantitative predictions of implant survival time, clinical service life, or actual intraoral fatigue performance. The additional 30° oblique loading conditions were not incorporated into the exploratory fatigue analysis.

### 2.7 *In vitro* displacement experiment for trend-level comparison

An *in vitro* displacement experiment was performed to descriptively compare inclination-related displacement patterns between the physical specimens and finite element models under the vertical loading condition. Synthetic bone blocks (Sawbones®; Pacific Research Laboratories, Vashon Island, WA, USA) with a density of 0.32 g/cm³ and an elastic modulus of 300–500 MPa were used to simulate type II mandibular bone. Each block was prepared with dimensions of 15 mm (mesiodistal) × 15 mm (buccolingual) × 25 mm (occlusoapical), corresponding to the simplified mandibular geometry used in the finite element models.

Implant osteotomies were prepared according to the manufacturer's drilling protocol, and the implants were inserted until the implant neck was level with the upper surface of the block. The final insertion torque was controlled at 25–35 N·cm to improve consistency among specimens. Physical assemblies corresponding to the two implant systems and five inclination angles were fabricated based on the same digital designs used for the finite element models. Five independent specimens were prepared for each implant system and inclination angle.

Mechanical testing was performed using a universal testing machine (ZwickRoell Z020, ZwickRoell GmbH & Co. KG, Ulm, Germany). Before testing, the machine was calibrated according to the manufacturer's instructions. Each specimen was fixed in a custom holding device to maintain the same orientation as the corresponding finite element model. A hemispherical loading nose with a diameter of 6 mm was positioned at the central fossa of the crown. After applying a preload of 5–10 N to establish stable contact, vertical loading was applied to 150 N at a crosshead speed of 1.0 mm/min. The load was applied parallel to the prosthetic crown axis, consistent with the vertical finite element loading condition.

Displacement was recorded from the universal testing machine output and defined as the vertical displacement of the crown loading point along the direction of the applied force. To ensure comparability, the finite element displacement value was extracted from the same crown central-fossa loading point and in the same direction. The mean displacement of the five specimens for each inclination angle was used for descriptive comparison with the corresponding finite element prediction.

Because the finite element models assumed ideal osseointegration and fully bonded interfaces, whereas the physical specimens involved implants mechanically inserted into synthetic bone blocks, the two conditions were not mechanically equivalent. Therefore, the *in vitro* experiment was used only for descriptive comparison of inclination-related displacement patterns between the physical and computational models. It was not used to validate the finite element model, assess mesh independence, or evaluate the response under the additional oblique loading condition.

### 2.8 Data presentation and interpretation

This study was designed as an exploratory and predominantly descriptive biomechanical investigation. Finite element outcomes were summarized according to stress-distribution patterns, peak stress values, mesh-convergence behavior, inclination-related trends, and differences between the vertical and oblique loading conditions. No inferential statistical testing was applied to the finite element outputs.

For each finite element outcome, the relative change associated with the oblique loading condition was calculated as follows:

$$\begin{array}{rl} \mathrm{Relative}\;\mathrm{change}= & (\mathrm{Oblique}-\mathrm{load}\;\mathrm{value}-\mathrm{Vertical}-\mathrm{load}\;\mathrm{value})/\\ & \mathrm{Vertical}-\mathrm{load}\;\mathrm{value}\times 100\text{\%}. \end{array}$$

For the *in vitro* displacement experiment, vertical displacement under a 150 N load was expressed as mean ± standard deviation. The experimental results were compared descriptively with the finite element displacement patterns across inclination angles and implant systems. No inferential statistical testing was performed, and the *in vitro* experiment was not treated as validation or numerical verification of the finite element model.

## 3 Results

### 3.1 Mesh convergence and implant stress distribution under vertical loading

The final meshes used for the main finite element analysis contained approximately 1.46–1.51 million nodes and 1.03–1.06 million elements across the ten models, with detailed node and element counts provided in Table 2. Mesh convergence was evaluated using the 0° and 20° configurations of both implant systems at three mesh densities.

**Table 2:** Number of nodes and elements for the model of the Ti-Zr implant.

Implant		Inclination angle				
		0°	5°	10°	15°	20°
Ti–Zr	Number of nodes	1,465,299	1,473,102	1,466,036	1,467,650	1,478,110
	Number of elements	1,027,639	1,033,965	1,028,327	1,029,829	1,033,086
**Ti**	Number of nodes	1,478,269	1,491,734	1,490,962	1,488,779	1,506,864
	Number of elements	1,034,410	1,047,733	1,044,194	1,046,136	1,056,529

For implant peak von Mises stress, the percentage changes between the coarse and medium meshes ranged from 3.20% to 3.68%, whereas the changes between the medium and fine meshes ranged from 1.61% to 1.85%. For cortical bone maximum principal stress, the corresponding changes ranged from 3.52% to 3.73% and from 1.76% to 2.08%, respectively. For cancellous bone maximum principal stress, the coarse-to-medium changes ranged from 3.47% to 4.11%, and the medium-to-fine changes ranged from 1.93% to 2.67%. Thus, all primary stress outcomes showed less than 5% change between the medium and fine meshes, indicating acceptable numerical stability under the present modeling conditions. Detailed convergence results are provided in Supplementary Table S1.

**Figure 2:**
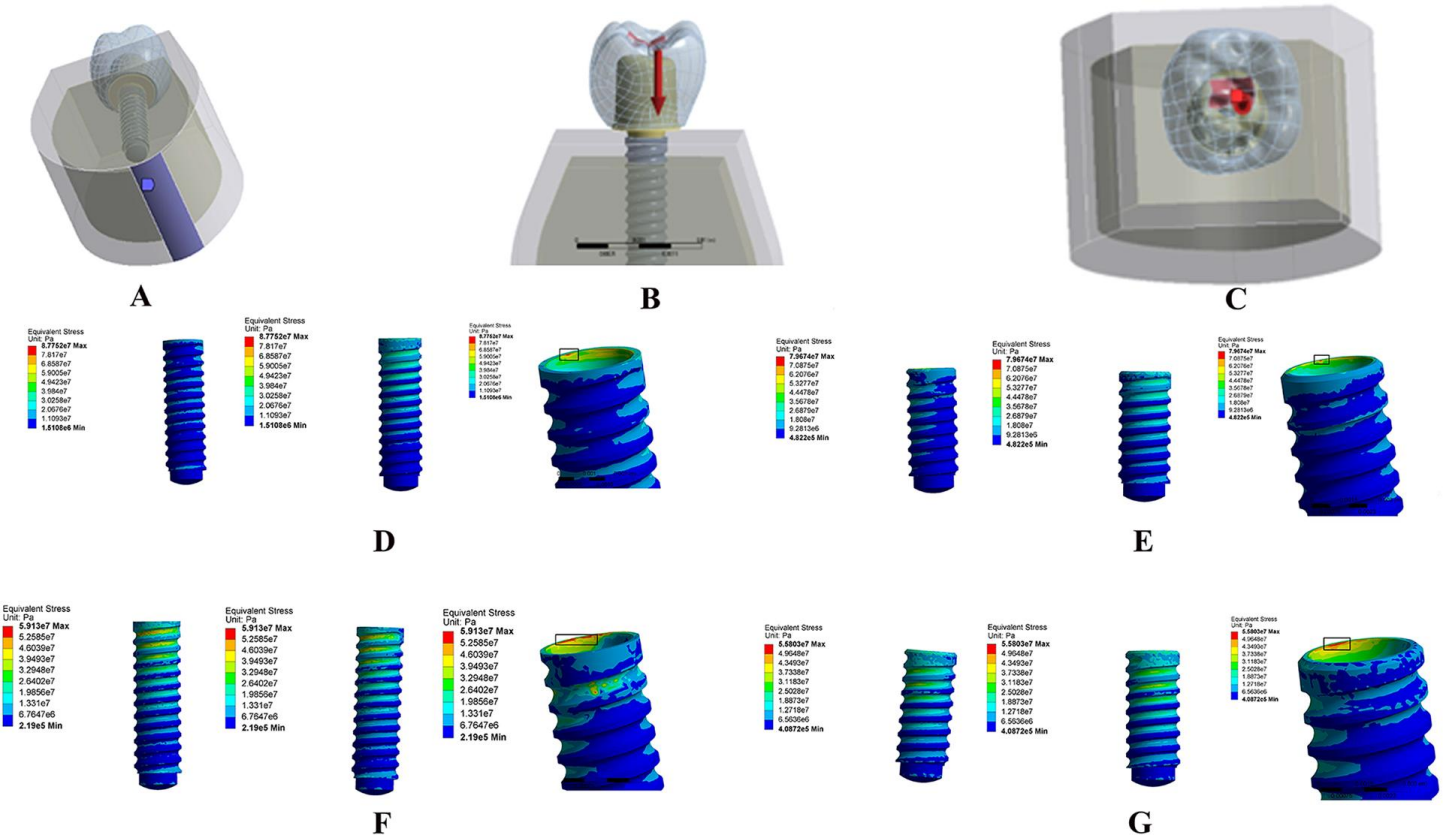
**(A)** Boundary conditions of the mandibular bone. **(B)** and **(C)** Loading conditions schematic. From left to right: overall stress distribution of the implant, buccal stress distribution of the implant, and maximum von Mises stress distribution of the implant. **(D)** 0° Ti–Zr implant model; **(E)** 0° pure titanium implant model; **(F)** 5° Ti–Zr implant model; **(G)** 5° pure titanium implant model.

**Figure 3:**
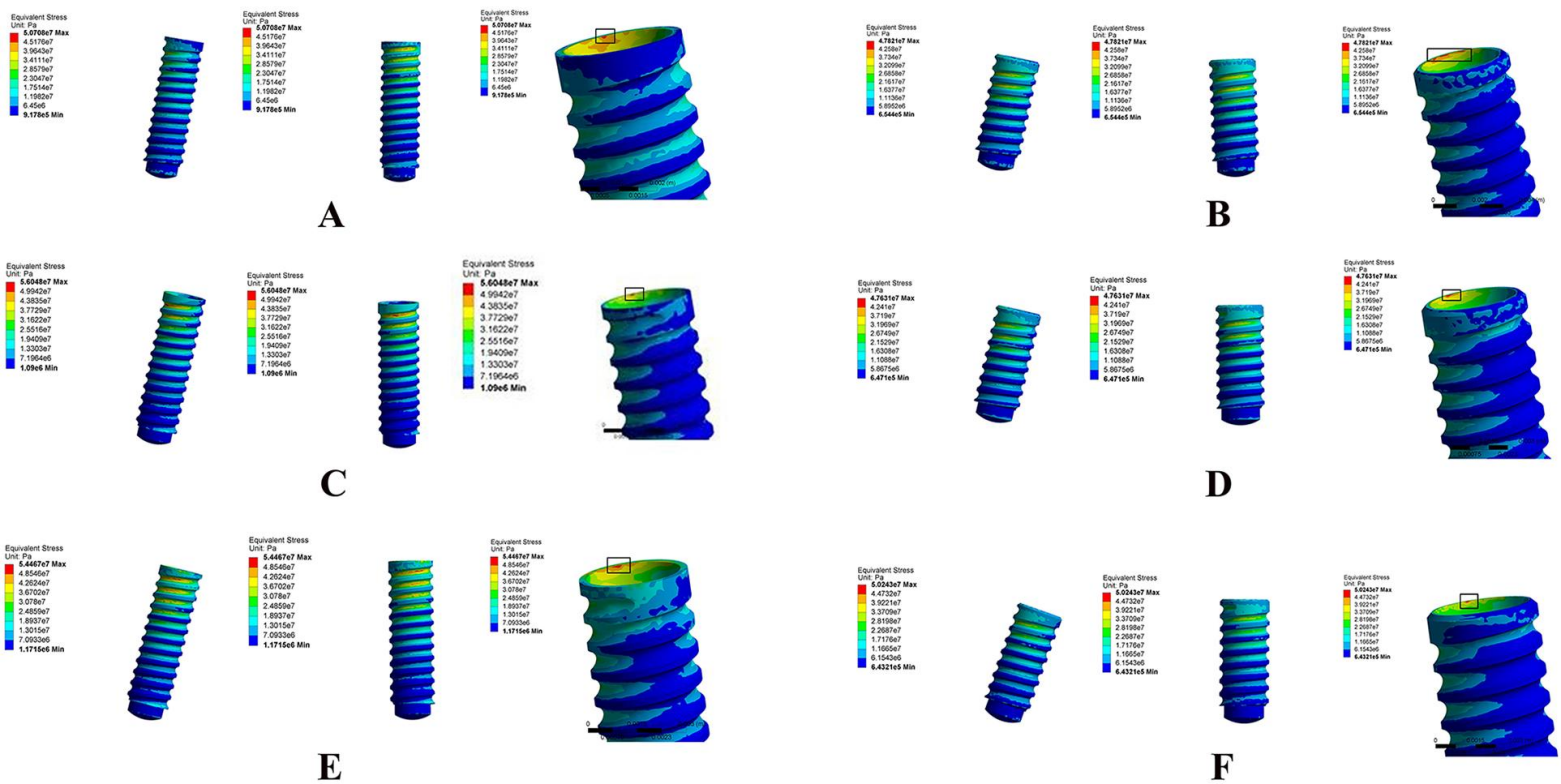
From left to right: overall stress distribution of the implant, buccal stress distribution of the implant, and maximum von Mises stress distribution of the implant. **(A)** 10° Ti–Zr implant model; **(B)** 10° pure titanium implant model; **(C)** 15° Ti–Zr implant model; **(D)** 15° pure titanium implant model; **(E)** 20° Ti–Zr implant model; **(F)** 20° pure titanium implant model.

Under vertical loading, von Mises stress was mainly concentrated at the implant neck and buccal coronal one-third region in both implant systems. The highest von Mises stress was consistently located at the inner buccal aspect of the implant neck (Figures 2, 3). The peak stress values are summarized in Table 3 and were used primarily to compare inclination-related stress-distribution patterns within the present models.

**Table 3:** Maximum equivalent stress of the implants (MPa).

Implant		Inclination angle				
		0°	5°	10°	15°	20°
Ti–Zr	Maximum	87.75	59.13	50.70	56.04	54.46
Ti	Maximum	79.67	55.80	47.82	47.63	50.24

For the 3.3 × 12 mm Ti–Zr implant system, peak von Mises stress decreased from 87.75 MPa at 0° to 50.70 MPa at 10°, followed by a slight increase to 56.04 MPa at 15° and 54.46 MPa at 20°. For the 4.1 × 10 mm titanium implant system, the corresponding value decreased from 79.67 MPa at 0° to 47.82 MPa at 10°, remained similar at 15° (47.63 MPa), and increased slightly to 50.24 MPa at 20°. Therefore, under vertical loading, both implant systems showed lower peak implant stress at 10° than at 0°, whereas the results at 15° and 20° were characterized by modest local fluctuations rather than a uniform monotonic increase.

Because the two implant configurations differed simultaneously in material, diameter, length, and geometry, the observed differences between the systems should be interpreted strictly as system-level findings.

### 3.2 Peri-implant bone stress distribution under vertical loading

**Figure 4:**
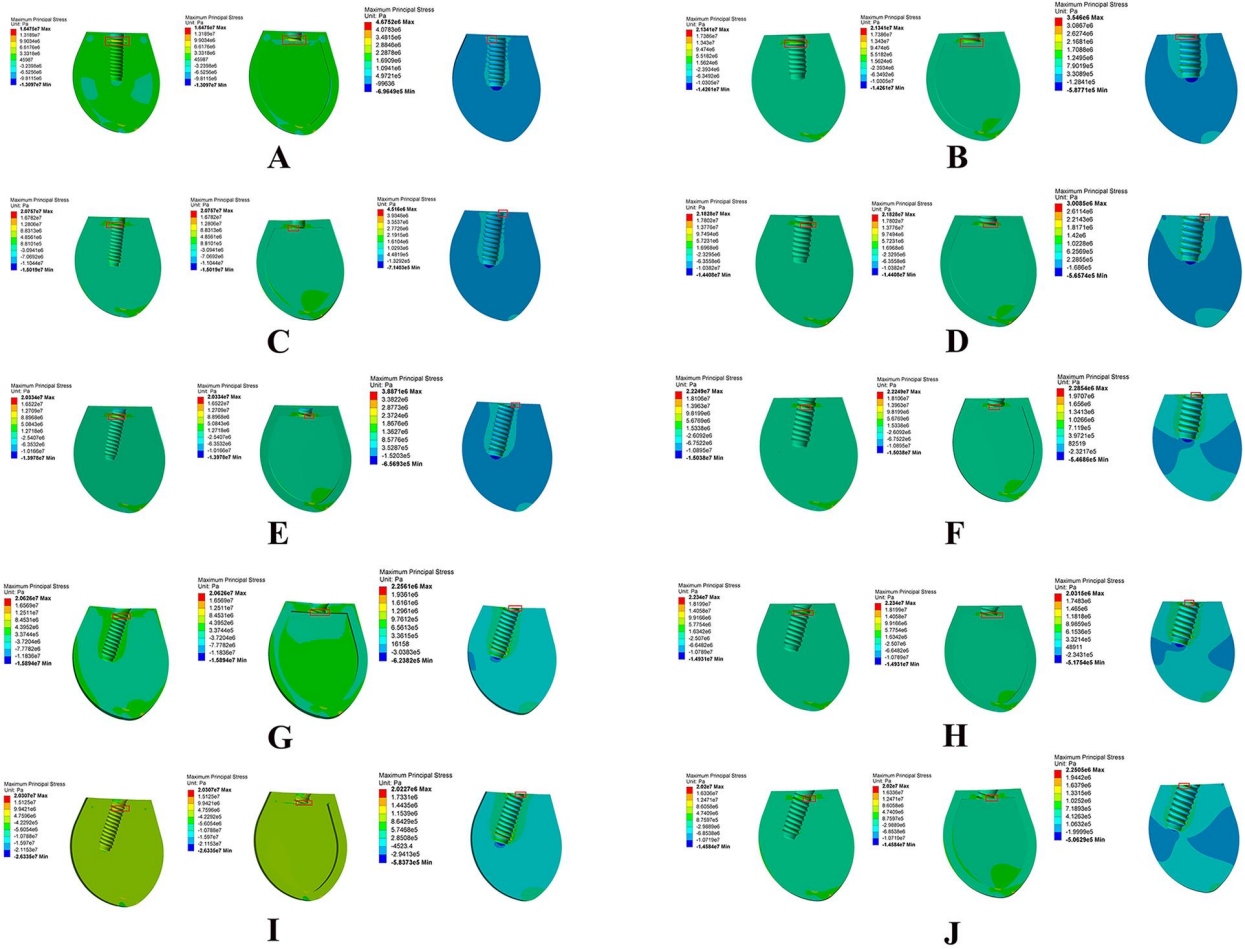
From left to right: maximum principal stress distribution of the entire mandibular bone, cortical bone, and cancellous bone. **(A)** 0° Ti–Zr implant model; **(B)** 0° pure titanium implant model; **(C)** 5° Ti–Zr implant model; **(D)** 5° pure titanium implant model; **(E)** 10° Ti–Zr implant model; **(F)** 10° pure titanium implant model; **(G)** 15° Ti–Zr implant model; **(H)** 15° pure titanium implant model; **(I)** 20° Ti–Zr implant model; **(J)** 20° pure titanium implant model.

Maximum principal stress in bone was evaluated separately in the cortical and cancellous bone regions. The reported values represent the peak tensile principal stress extracted from the predefined peri-implant cortical and cancellous regions. Under vertical loading, higher stress concentration was generally observed in the coronal cortical bone than in the cancellous bone (Figure 4; Table 4).

**Table 4:** Maximum principal stress of bone (MPa).

Bone	Inclination angle	Ti–Zr	Ti
Cortical bone	0°	16.47	21.34
	5°	19.87	21.82
	10°	20.33	22.24
	15°	20.62	22.34
	20°	20.30	20.20
Cancellous bone	0°	4.67	3.54
	5°	4.51	3.00
	10°	3.88	2.28
	15°	2.25	2.03
	20°	2.02	2.25

For the Ti–Zr implant system, cortical bone maximum principal stress increased from 16.47 MPa at 0° to 19.87 MPa at 5°, 20.33 MPa at 10°, and 20.62 MPa at 15°, followed by a slight decrease to 20.30 MPa at 20°. For the titanium implant system, cortical bone stress increased from 21.34 MPa at 0° to 21.82 MPa at 5°, 22.24 MPa at 10°, and 22.34 MPa at 15°, and then decreased to 20.20 MPa at 20°. Therefore, cortical bone stress increased between 0° and 15° in both systems but did not show a continuous increase at 20°.

For cancellous bone, the Ti–Zr implant system showed a gradual decrease from 4.67 MPa at 0° to 4.51 MPa at 5°, 3.88 MPa at 10°, 2.25 MPa at 15°, and 2.02 MPa at 20°. In the titanium implant system, cancellous bone stress decreased from 3.54 MPa at 0° to 3.00 MPa at 5°, 2.28 MPa at 10°, and 2.03 MPa at 15°, followed by a slight increase to 2.25 MPa at 20°.

These findings indicate that cortical and cancellous bone exhibited different inclination-related responses under vertical loading. Accordingly, peri-implant bone stress should not be summarized as a single uniform trend.

### 3.3 Additional oblique loading analysis

Under the additional 150 N load applied at 30° to the prosthetic crown axis in the buccolingual plane, implant and peri-implant bone stresses were higher than those observed under vertical loading in all configurations (Table 5).

**Table 5:** Comparison of the primary finite element stress outcomes under vertical and 30° oblique loading conditions.

Implant system	Lingual inclination	Implant peak von Mises stress under vertical load (MPa)	Implant peak von Mises stress under 30° oblique load (MPa)	Change (%)	Cortical bone maximum principal stress under vertical load (MPa)	Cortical bone maximum principal stress under 30° oblique load (MPa)	Change (%)	Cancellous bone maximum principal stress under vertical load (MPa)	Cancellous bone maximum principal stress under 30° oblique load (MPa)	Change (%)
Ti–Zr, 3.3 × 12 mm	0°	87.75	124.61	42.0	16.47	22.23	35.0	4.67	5.84	25.1
Ti–Zr, 3.3 × 12 mm	5°	59.13	88.70	50.0	19.87	28.22	42.0	4.51	5.95	31.9
Ti–Zr, 3.3 × 12 mm	10°	50.70	82.13	62.0	20.33	30.50	50.0	3.88	5.43	39.9
Ti–Zr, 3.3 × 12 mm	15°	56.04	98.07	75.0	20.62	32.99	60.0	2.25	3.38	50.2
Ti–Zr, 3.3 × 12 mm	20°	54.46	102.38	88.0	20.30	34.92	72.0	2.02	3.27	61.9
Ti, 4.1 × 10 mm	0°	79.67	109.95	38.0	21.34	28.81	35.0	3.54	4.43	25.1
Ti, 4.1 × 10 mm	5°	55.80	82.03	47.0	21.82	30.98	42.0	3.00	3.96	32.0
Ti, 4.1 × 10 mm	10°	47.82	75.55	58.0	22.24	33.36	50.0	2.28	3.19	39.9
Ti, 4.1 × 10 mm	15°	47.63	81.45	71.0	22.34	35.74	60.0	2.03	3.05	50.2
Ti, 4.1 × 10 mm	20°	50.24	92.44	84.0	20.20	34.74	72.0	2.25	3.65	62.2

A static load of 150 N was applied either parallel to the prosthetic crown axis or at 30° to the prosthetic crown axis in the buccolingual plane. Percentage change was calculated as: [(oblique-load value − vertical-load value)/vertical-load value] × 100%.

For the Ti–Zr implant system, implant peak von Mises stress values under oblique loading were 124.61 MPa at 0°, 88.70 MPa at 5°, 82.13 MPa at 10°, 98.07 MPa at 15°, and 102.38 MPa at 20°. Compared with the corresponding vertical-load values, these represented increases of 42.0%, 50.0%, 62.0%, 75.0%, and 88.0%, respectively. Although implant stress remained lower at 10° than at 0°, the proportional increase associated with oblique loading became progressively larger as the inclination angle increased.

For the titanium implant system, implant peak von Mises stress values under oblique loading were 109.95 MPa at 0°, 82.03 MPa at 5°, 75.55 MPa at 10°, 81.45 MPa at 15°, and 92.44 MPa at 20°. These values were 38.0%, 47.0%, 58.0%, 71.0%, and 84.0% higher, respectively, than those obtained under vertical loading. As in the Ti–Zr system, peak implant stress remained lower at 10° than at 0°, but the relative amplification under oblique loading was greater at the larger inclination angles.

Cortical bone maximum principal stress also increased under oblique loading. In the Ti–Zr system, cortical bone stress values were 22.23 MPa at 0°, 28.22 MPa at 5°, 30.50 MPa at 10°, 32.99 MPa at 15°, and 34.92 MPa at 20°. These represented increases of 35.0%–72.0% relative to vertical loading. In the titanium system, the corresponding values were 28.81 MPa, 30.98 MPa, 33.36 MPa, 35.74 MPa, and 34.74 MPa, also corresponding to increases of 35.0%–72.0%. Unlike implant von Mises stress, cortical bone stress increased from 0° to 10° in both systems under the oblique loading condition.

Cancellous bone maximum principal stress under oblique loading was 5.84 MPa, 5.95 MPa, 5.43 MPa, 3.38 MPa, and 3.27 MPa at 0°, 5°, 10°, 15°, and 20°, respectively, in the Ti–Zr system. In the titanium system, the corresponding values were 4.43 MPa, 3.96 MPa, 3.19 MPa, 3.05 MPa, and 3.65 MPa. Compared with vertical loading, cancellous bone stress increased by approximately 25.1%–61.9% in the Ti–Zr system and by 25.1%–62.2% in the titanium system.

Overall, the additional oblique loading analysis showed that the inclination-related response depended on the evaluated component. The lower implant von Mises stress observed at 10° than at 0° was maintained under oblique loading in both systems. However, cortical bone maximum principal stress increased across the same inclination range. Furthermore, the relative stress amplification produced by oblique loading was generally greater at 15° and 20°. Therefore, the 0°–10° tendency was only partially maintained and should not be interpreted as a uniformly favorable biomechanical response across all outcomes.

### 3.4 Fatigue analysis under the vertical loading protocol

**Figure 5:**
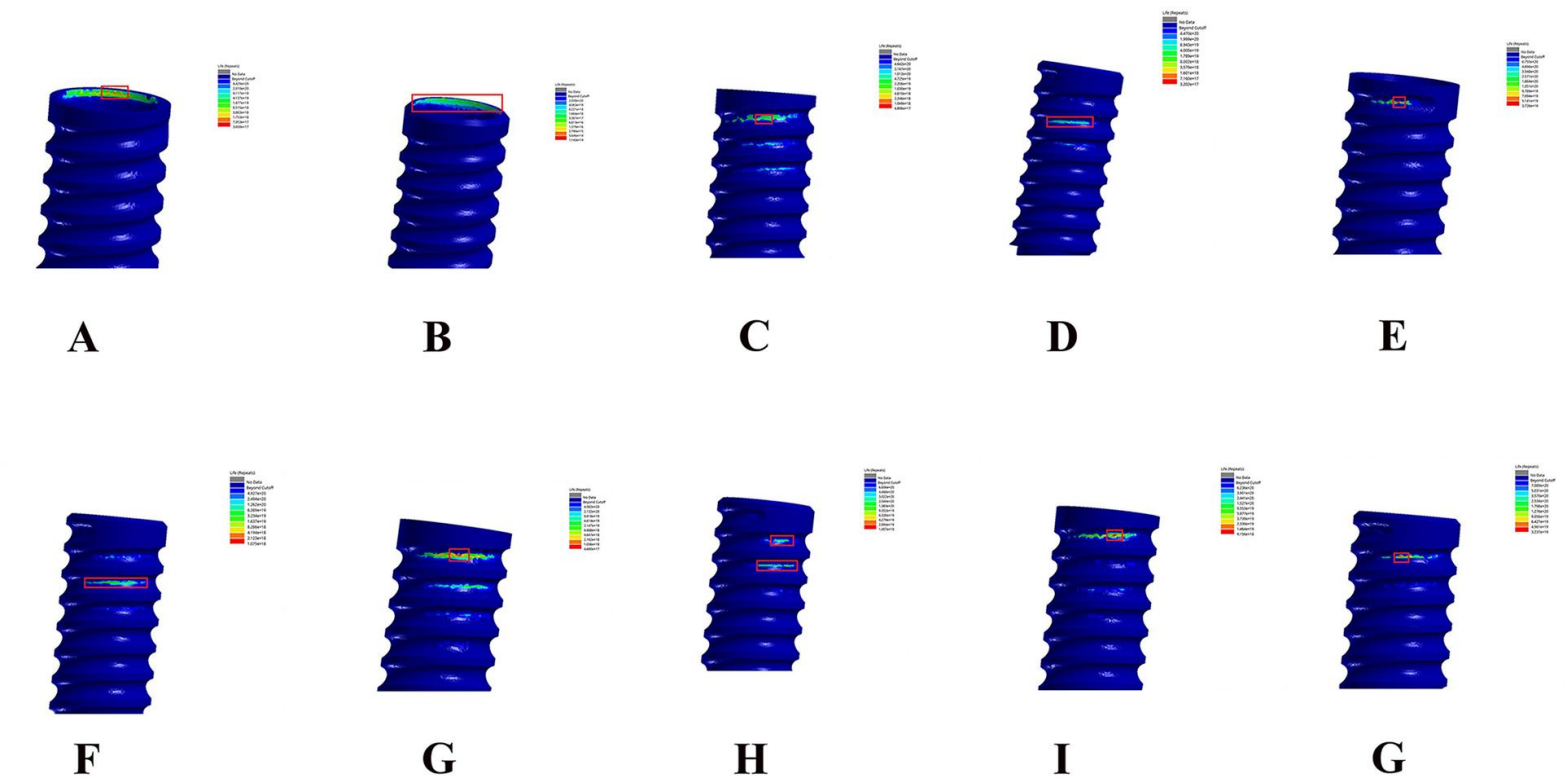
Fatigue analysis results. **(A)** 3.3 × 12 mm Ti–Zr implant with 0° inclination; **(B)** 4.1 × 10 mm pure titanium implant with 0° inclination; **(C)** 3.3 × 12 mm Ti–Zr implant with 5° inclination; **(D)** 4.1 × 10 mm pure titanium implant with 5° inclination; **(E)** 3.3 × 12 mm Ti–Zr implant with 10° inclination; **(F)** 4.1 × 10 mm pure titanium implant with 10° inclination; **(G)** 3.3 × 12 mm Ti–Zr implant with 15° inclination; **(H)** 4.1 × 10 mm pure titanium implant with 15° inclination; **(I)** 3.3 × 12 mm Ti–Zr implant with 20° inclination; **(J)** 4.1 × 10 mm pure titanium implant with 20° inclination.

The fatigue analysis identified the inner buccal aspect of the implant neck as the fatigue-critical region in the 0° models. In the 5°, 10°, 15°, and 20° models, the fatigue-critical region shifted mainly toward the buccal coronal one-third of the implant (Figure 5).

The exploratory software-estimated fatigue-cycle outputs for both implant systems are summarized in Table 6. Under the simplified vertical cyclic loading protocol and software-estimated fatigue settings, all models showed high calculated cycle numbers. These values should be interpreted only as exploratory comparative fatigue tendencies under the present numerical assumptions and not as predictions of clinical implant survival, actual intraoral service life, or long-term clinical performance. The additional 30° oblique loading condition was not included in the exploratory fatigue analysis.

**Table 6:** Exploratory software-estimated fatigue-cycle outputs under the simplified vertical cyclic loading protocol.

Implant	Inclination angle	Number of iterations
Ti–Zr	0°	3.608 × 10^17^
	5°	4.897 × 10^17^
	10°	3.726 × 10^19^
	15°	3.379 × 10^19^
	20°	1.601 × 10^19^
Ti	0°	1.706 × 10^16^
	5°	3.203 × 10^17^
	10°	1.075 × 10^18^
	15°	1.958 × 10^19^
	20°	3.237 × 10^19^

### 3.5 *In vitro* displacement results under vertical loading

**Figure 6:**
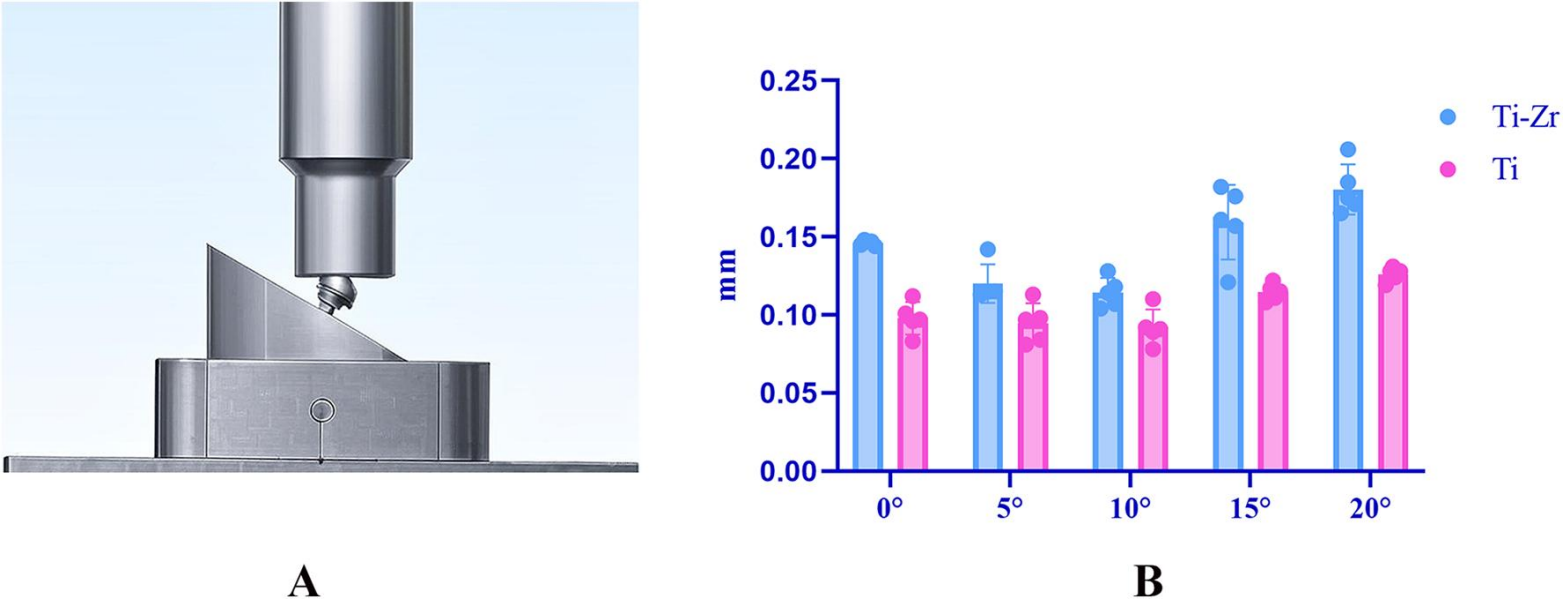
Descriptive *in-vitro* displacement comparison. **(A)** Schematic illustration of the experimental loading setup showing the vertical load applied to the crown central fossa parallel to the prosthetic crown axis. **(B)** Experimentally measured vertical displacement of the crown loading point at 0°, 5°, 10°, 15°, and 20° inclination angles for both implant systems. The experiment was used only to describe inclination-related displacement patterns and was not considered validation of the finite element model.

Under a 150 N vertical load, displacement of the crown loading point generally increased with larger inclination angles in both implant systems. For the titanium implant system, the mean displacements at 0°, 5°, 10°, 15°, and 20° were 0.0978 ± 0.0104 mm, 0.0946 ± 0.0127 mm, 0.0920 ± 0.0115 mm, 0.1146 ± 0.0054 mm, and 0.1260 ± 0.0046 mm, respectively. For the Ti–Zr implant system, the corresponding values were 0.1462 ± 0.0016 mm, 0.1200 ± 0.0123 mm, 0.1142 ± 0.0095 mm, 0.1594 ± 0.0238 mm, and 0.1804 ± 0.0161 mm, respectively (Figure 6).

For both implant systems, displacement remained relatively stable from 0° to 10° and increased more clearly at 15° and 20°. The Ti–Zr implant system showed larger displacement values than the titanium implant system at each inclination angle. Because the two implant systems differed in material, diameter, length, and geometry, these displacement differences should be interpreted as system-level observations rather than as isolated material- or diameter-specific effects.

The experimental and finite element results showed generally similar inclination-related displacement patterns under the vertical loading condition. However, the finite element models assumed ideal osseointegration and fully bonded interfaces, whereas the *in vitro* specimens involved implants mechanically inserted into synthetic bone blocks. Therefore, the two conditions were not mechanically equivalent. No inferential statistical analysis was performed for this comparison. Accordingly, the experimental findings are reported only as a descriptive comparison of displacement patterns and should not be interpreted as validation, numerical verification, or confirmation of the finite element model. The *in vitro* experiment did not evaluate the additional oblique loading condition.

## 4 Discussion

Posterior molar implantation is often complicated by high occlusal loading, alveolar ridge resorption, limited buccolingual bone width, and proximity to important anatomical structures. Although bone augmentation can improve local implant placement conditions, it may also increase surgical trauma, treatment duration, cost, and complication risk (3). In selected cases, inclined implant placement may help avoid anatomical constraints and increase the available implant length and bone contact area. However, when lingual inclination is combined with a narrow-diameter implant configuration, the resulting implant–bone complex should be regarded as an integrated biomechanical system rather than as the effect of a single isolated parameter.

The present study evaluated the biomechanical response of a 3.3 × 12 mm Ti–Zr implant system and a 4.1 × 10 mm commercially pure titanium implant system at different lingual inclination angles under vertical and representative oblique loading conditions. Under vertical loading, von Mises stress was mainly concentrated at the implant neck and buccal coronal one-third region in both systems. This finding is consistent with previous biomechanical studies identifying the cervical implant region as a mechanically vulnerable area under occlusal loading (28). Peak implant stress decreased from 0° to 10° and then showed modest increases or local fluctuations at 15° and 20°. This pattern suggests that, under a vertical crown-axis load, moderate lingual inclination did not necessarily increase implant-body stress within the present model.

The additional oblique loading analysis demonstrated that loading direction materially influenced both stress magnitude and the inclination-related response. Under the 30° oblique load, implant and peri-implant bone stresses increased in all configurations compared with vertical loading. The relative increase in implant peak von Mises stress ranged from 42.0% to 88.0% in the Ti–Zr system and from 38.0% to 84.0% in the titanium system. The proportional stress amplification was generally greater at 15° and 20°, indicating that the larger inclination configurations were more sensitive to non-axial loading. This finding is biomechanically plausible because the horizontal force component increases the bending moment acting on the implant–abutment complex and peri-implant cortical bone.

Despite the overall increase in stress under oblique loading, implant peak von Mises stress remained lower at 10° than at 0° in both systems. However, this pattern was not reproduced uniformly across all evaluated outcomes. Cortical bone maximum principal stress increased from 0° to 10° under both vertical and oblique loading, whereas cancellous bone showed a different inclination-related pattern. Accordingly, the 0°–10° tendency observed for implant-body stress should not be generalized to the entire implant–bone system. These findings emphasize that implant stress, cortical bone stress, and cancellous bone stress reflect different aspects of load transfer and should be interpreted separately. A configuration associated with lower implant-body stress may not necessarily reduce tensile stress in the surrounding cortical bone.

Differences between the two implant systems should also be interpreted cautiously. The two configurations differed simultaneously in implant material, diameter, length, and geometry; therefore, the present study cannot determine the independent contribution of Ti–Zr alloy, implant diameter, implant length, or macrogeometry. The observed differences in peak stress and displacement should instead be regarded as system-level observations under identical inclination and loading conditions. They should not be attributed directly to the Ti–Zr alloy, narrower diameter, greater length, or any other individual design feature. Previous biomechanical studies have similarly shown that implant geometry, material properties, bone quality, and loading direction interact to determine peri-implant stress distribution and mechanical behavior (12, 29, 30). A factorial design in which one parameter is varied while the others are held constant would be required to isolate the effect of each design variable.

At the implant–bone interface, maximum principal stress was predominantly concentrated in the cortical bone, particularly in the coronal peri-implant region. This is consistent with the load-bearing role of crestal cortical bone around dental implants (28). Under vertical loading, cortical bone stress generally increased from 0° to 15° and then decreased slightly at 20° in both systems. Cancellous bone stress, in contrast, decreased progressively in the Ti–Zr system and showed a non-monotonic pattern in the titanium system. Under oblique loading, cortical and cancellous bone stresses increased in all configurations, but their inclination-related patterns remained different. The greater increase in cortical bone stress under non-axial loading highlights the importance of the coronal cortical region in resisting bending-related loads. These findings support separate interpretation of cortical and cancellous bone outcomes rather than use of a single generalized “bone stress” response. The multifactorial nature of peri-implant stress distribution, including implant geometry, peri-implant bone thickness, inclination, structural rigidity, and loading direction, has also been emphasized in previous studies (6, 7, 9, 10, 12, 13).

The additional oblique loading analysis improves the biomechanical relevance of the present study by showing that the vertical-load results cannot be interpreted independently of loading direction. Nevertheless, the additional analysis should be regarded as a representative sensitivity assessment rather than a complete simulation of posterior mastication. Only one 30° load applied in the buccolingual plane at a fixed central-fossa contact point was evaluated. In clinical function, the direction, magnitude, and point of application of occlusal force change continuously during chewing and lateral excursions. Mesiodistal force components, multiple contact points, parafunctional loading, impact, and variable-amplitude cyclic loading may produce different stress patterns. Therefore, the present oblique loading results extend the vertical-load comparison but do not fully reproduce the complex intraoral loading environment.

The *in vitro* displacement experiment provided a descriptive comparison with the computational displacement patterns under vertical loading. In both implant systems, displacement remained relatively stable from 0° to 10° and increased more clearly at 15° and 20°. Nevertheless, the finite element models assumed ideal osseointegration and fully bonded interfaces, whereas the experimental specimens relied on mechanical insertion into synthetic bone blocks. These conditions were not mechanically equivalent, and the experiment was not designed to establish numerical agreement or validate the computational model. The displacement findings therefore represent only a limited trend-level comparison. They do not assess mesh independence and do not confirm the response observed under the additional oblique loading conditions.

Fatigue analysis also requires cautious interpretation. Although the calculated cycle values suggested low relative fatigue damage under the simplified vertical cyclic loading protocol, these findings remain highly exploratory because implant-specific S–N data for Ti–Zr implants were limited and the fatigue parameters were estimated using available material data and software-based assumptions. The additional oblique load was not incorporated into the analysis of fatigue. This is important because non-axial loading may increase bending stress and alter fatigue-critical regions. Intraoral fatigue behavior is also influenced by variable-amplitude mastication, lateral and oblique loading, parafunctional activity, corrosion–fatigue interactions, implant–abutment micromotion, screw preload, and patient-specific occlusal conditions. Accordingly, the reported fatigue outputs should be interpreted only as comparative indicators under standardized numerical assumptions and not as predictions of clinical service life, long-term survival, or actual intraoral performance (31–33).

Several limitations should be acknowledged. First, the comparison involved two complete implant systems, and the independent effects of material, diameter, length, and geometry could not be isolated. Second, the finite element models used simplified mandibular geometry, type II bone properties, ideal osseointegration, and bonded implant–abutment and abutment–crown interfaces. Cortical and cancellous bone were modeled as homogeneous, isotropic, and linearly elastic materials, although biological bone is heterogeneous, anisotropic, and patient-specific. These assumptions may affect stress transfer by reducing interfacial micromotion, frictional sliding, contact-related stress concentration, and screw-joint effects. Third, although an additional 30° oblique load was included, only one non-axial loading direction and one fixed loading point were evaluated. The analysis did not include opposite buccolingual loading, mesiodistal loading, changing contact positions, parafunctional forces, impact-related loading, or multidirectional cyclic conditions. Fourth, angled abutments were used to maintain prosthetic alignment and comparability between groups; however, this configuration may have partially modified cervical and peri-implant stress concentration and may represent a more favorable biomechanical condition than some clinical scenarios. Fifth, mesh convergence was evaluated using representative 0° and 20° configurations rather than independently for every inclination model. Although the primary stress outcomes showed less than 5% change between the medium and fine meshes, some residual mesh sensitivity in highly localized peak stress regions cannot be completely excluded. Sixth, the *in vitro* displacement comparison was performed only under vertical loading and should be considered independently from the mesh convergence and oblique loading analyses. Finally, the findings remain model-derived biomechanical observations and should not be translated directly into clinical thresholds without patient-specific simulations, broader multidirectional loading analyses, sensitivity analyses, and long-term clinical validation.

Within these limitations, the present simplified finite element analysis provides an exploratory framework for comparing the relative biomechanical responses of the studied implant-system configurations under standardized vertical and representative oblique loading conditions. The additional loading analysis showed that the inclination-related response was outcome-dependent. Implant peak von Mises stress remained lower at 10° than at 0° under both loading conditions, whereas cortical bone maximum principal stress increased across the same inclination range. Larger inclination angles also showed greater proportional stress amplification under oblique loading. Therefore, the findings do not identify a uniformly favorable inclination range but instead demonstrate that biomechanical interpretation depends on the evaluated component and loading direction. The *in vitro* displacement experiment and exploratory fatigue estimation provide separate supplementary observations and should not be interpreted as validation of the computational model, demonstration of clinical feasibility, or evidence supporting a clinical inclination threshold or surgical recommendation (34–37).

## 5 Conclusion

Within the limitations of this simplified finite element and *in vitro* biomechanical study, the investigated implant systems showed loading- and outcome-dependent inclination-related responses. The additional 30° oblique load increased implant and peri-implant bone stresses in all configurations, with greater proportional stress amplification generally observed at larger inclination angles. In both implant systems, implant peak von Mises stress remained lower at 10° than at 0° under both vertical and oblique loading conditions. However, cortical bone maximum principal stress increased across the same inclination range, indicating that the 0°–10° response was not uniformly favorable across all evaluated biomechanical outcomes. Therefore, this range should not be interpreted as a clinically validated threshold or recommended inclination range.

Because the two implant configurations differed simultaneously in material, diameter, length, and geometry, the observed differences should be regarded strictly as system-level findings and should not be interpreted as evidence of the independent effect of any single design parameter. Further patient-specific biomechanical analyses and long-term clinical studies are required to determine whether these exploratory model-derived observations have clinical relevance; the present findings alone cannot establish an inclination threshold or support surgical recommendations.

## Data Availability

The original contributions presented in the study are included in the article/Supplementary Material, further inquiries can be directed to the corresponding author/s.
