# Supplementary material for: Biomechanical evaluation of narrow-diameter Ti–Zr implant systems at different lingual inclination angles in the molar region
Source: Front Oral Health. 2026 Jul 6;7:1878801. doi: 10.3389/froh.2026.1878801 (PMC13381632; doi:10.3389/froh.2026.1878801)
Supplement: Supplementary file 1 [file Table1.docx]

**Supplementary Table S1.** Mesh convergence analysis for representative implant-system configurations.

| **Implant system** | **Inclination** | **Mesh density** | **Nodes** | **Elements** | **Implant peak von Mises stress (MPa)** | **Change from previous mesh (%)** | **Cortical bone maximum principal stress (MPa)** | **Change from previous mesh (%)** | **Cancellous bone maximum principal stress (MPa)** | **Change from previous mesh (%)** |
| --- | --- | --- | --- | --- | --- | --- | --- | --- | --- | --- |
| Ti–Zr, 3.3 × 12 mm | 0° | Coarse | 682,450 | 468,320 | 83.16 | — | 15.61 | — | 4.42 | — |
| Ti–Zr, 3.3 × 12 mm | 0° | Medium | 1,042,680 | 721,540 | 86.34 | 3.68 | 16.18 | 3.52 | 4.58 | 3.49 |
| Ti–Zr, 3.3 × 12 mm | 0° | Fine | 1,465,299 | 1,027,639 | 87.75 | 1.61 | 16.47 | 1.76 | 4.67 | 1.93 |
| Ti–Zr, 3.3 × 12 mm | 20° | Coarse | 705,820 | 486,410 | 51.63 | — | 19.18 | — | 1.89 | — |
| Ti–Zr, 3.3 × 12 mm | 20° | Medium | 1,071,460 | 742,680 | 53.47 | 3.44 | 19.89 | 3.57 | 1.97 | 4.06 |
| Ti–Zr, 3.3 × 12 mm | 20° | Fine | 1,478,110 | 1,033,086 | 54.46 | 1.82 | 20.30 | 2.02 | 2.02 | 2.48 |
| Ti, 4.1 × 10 mm | 0° | Coarse | 697,540 | 479,860 | 75.68 | — | 20.13 | — | 3.34 | — |
| Ti, 4.1 × 10 mm | 0° | Medium | 1,063,280 | 735,420 | 78.24 | 3.27 | 20.91 | 3.73 | 3.46 | 3.47 |
| Ti, 4.1 × 10 mm | 0° | Fine | 1,478,269 | 1,034,410 | 79.67 | 1.80 | 21.34 | 2.01 | 3.54 | 2.26 |
| Ti, 4.1 × 10 mm | 20° | Coarse | 721,630 | 498,730 | 47.73 | — | 19.06 | — | 2.10 | — |
| Ti, 4.1 × 10 mm | 20° | Medium | 1,096,820 | 762,350 | 49.31 | 3.20 | 19.78 | 3.64 | 2.19 | 4.11 |
| Ti, 4.1 × 10 mm | 20° | Fine | 1,506,864 | 1,056,529 | 50.24 | 1.85 | 20.20 | 2.08 | 2.25 | 2.67 |

Percentage change was calculated as:

Percentage change = |Resultfiner − Resultcoarser| / Resultfiner × 100%.

Across the four representative configurations, the percentage changes between the medium and fine meshes ranged from 1.61% to 1.85% for implant peak von Mises stress, from 1.76% to 2.08% for cortical bone maximum principal stress, and from 1.93% to 2.67% for cancellous bone maximum principal stress. All changes were below the predefined 5% convergence criteria.
